# Molecular and computational approaches to map regulatory elements in 3D chromatin structure

**DOI:** 10.1186/s13072-021-00390-y

**Published:** 2021-03-19

**Authors:** Beoung Hun Lee, Suhn K. Rhie

**Affiliations:** Department of Biochemistry and Molecular Medicine and the Norris Comprehensive Cancer Center, Keck School of Medicine, University of Southern California, Los Angeles, CA 90089 USA

**Keywords:** Epigenomics, Regulatory elements, Chromatin interactions, Databases, Analysis tools

## Abstract

Epigenetic marks do not change the sequence of DNA but affect gene expression in a cell-type specific manner by altering the activities of regulatory elements. Development of new molecular biology assays, sequencing technologies, and computational approaches enables us to profile the human epigenome in three-dimensional structure genome-wide. Here we describe various molecular biology techniques and bioinformatic tools that have been developed to measure the activities of regulatory elements and their chromatin interactions. Moreover, we list currently available three-dimensional epigenomic data sets that are generated in various human cell types and tissues to assist in the design and analysis of research projects.

## Background

**Fig. 1:**
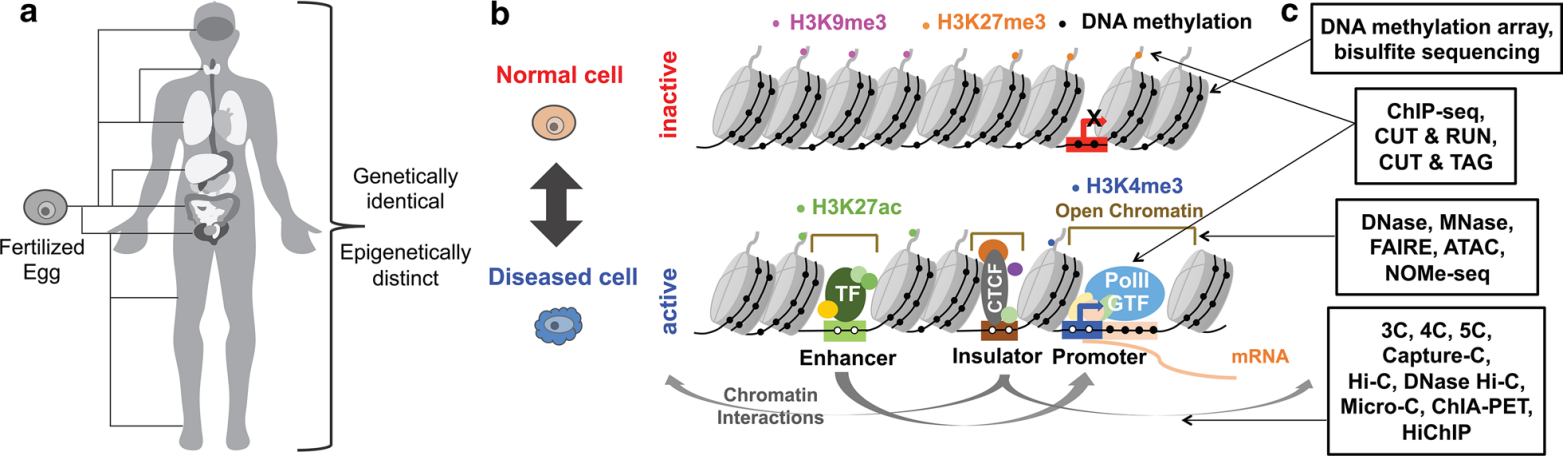
Overview of epigenome change in the human genome. **a** Human cells within an individual are genetically identical across all cell types, but distinct epigenome profiles are detected between cell types. **b** Epigenome changes when normal cells become diseased cells, and vice versa. DNA methylation, histone marks H3K9me3 (heterochromatin region) and H3K27me3 (repressed region) are usually associated with inactive, closed chromatin, while unmethylated DNA, histone marks H3K4me3 (active promoter) and H3K27ac (active enhancer), and transcription factor (TF) binding are found in active, open chromatin. An insulator marked by CTCF can act as a barrier to prevent enhancer-promoter interaction and decrease the rate of transcription or stop the spreading of heterochromatin to increase rate of transcription. **c** DNA methylation arrays and bisulfite sequencing are used to measure DNA methylation levels. ChIP-seq, CUT & RUN, and CUT & TAG are used to identify regulatory elements using histone mark and TF enriched regions. 3C, 4C, 5C, Capture-C, Hi-C, DNase Hi-C, ChIA-PET, and HiChIP are used to map chromatin interactions. PolII: RNA Polymerase II, GTF: General transcription factor

Nearly every cell in the human body has the same DNA. However, each cell has a distinct gene expression profile. The cell-type specific gene expression patterns come from differences in the epigenome (Fig. 1a). The epigenome is a collection of sequence-independent regulatory modifications to DNA or protein, which include, but are not limited to histone modifications, DNA methylation, and chromatin organization [1]. Histones are proteins that tightly wrap and pack DNA into nucleosomes, and their modifications are associated with the chromatin states [2, 3]. Chromatin states are largely divided into two states: inactive chromatin and active chromatin. Heterochromatin is a form of chromatin that is densely packed and transcriptionally inactive. Heterochromatin regions are marked by histone modification H3K9me3. Inactive chromatin regions also include cis-regulatory elements (e.g., promoters, enhancers, insulators) that are silenced and repressed. These repressed regions are marked by histone modification H3K27me3. DNA methylation, the addition of a methyl group to the cytosine of CpG, is often found in inactive regulatory elements, where their target genes are repressed [4] (Fig. 1b, top). On the other hand, euchromatin is the transcriptionally active form of chromatin. Active regions of chromatin include regulatory elements that are open and accessible for proteins to bind. Regulatory elements bound by transcription factors (TFs) control the rate of transcription [5]. A promoter is located near the transcriptional start site (TSS) of a target gene, and an active promoter is unmethylated and marked by histone modification H3K4me3 [6]. An enhancer, marked by histone modification H3K4me1 for poised and H3K27ac for active status, is located distal to the TSS of a target gene [7]. Enhancers interact with the promoter of a target gene to increase the rate of transcription. An insulator, which is marked by CTCF (CCCTC-binding factor), can either decrease the rate of transcription by interfering with the promoter-enhancer interaction or increase the transcription by acting as a barrier to stop the spread of heterochromatin [8] (Fig. 1b, bottom). The most likely model that has been suggested for explaining the mechanisms by which regulatory elements influence gene expression is a looping model. In a looping model, TFs bring regulatory elements into proximity by forming a loop [9]. For example, forming promoter and enhancer loops increases the expression of a target gene [10]. Insulators also form a loop, often preventing an enhancer located between insulators from interacting with the promoter of a non-target gene [11].

Chromatin states and interactions not only change among cell types but also change between inactive and active status when normal cells become diseased cells, and vice versa (Fig. 1b). Dysregulation of the human epigenome can result in cancer, autoimmune diseases, psychiatric diseases, and many more [12–14]. For example, it is reported that changes in DNA methylation of CTCF binding sites result in the loss of insulators and promote chromatin interactions between enhancers and oncogenes in tumors [15]. Profiling and characterizing three-dimensional (3D) epigenomes is crucial to understanding of underlying molecular mechanisms and promoting future development of treatments.

The development of molecular biology techniques coupled with next generation sequencing now enables us to map epigenomes genome-wide. For example, ChIP-seq (Chromatin immunoprecipitation sequencing), CUT & RUN (Cleavage under targets and release using nuclease) sequencing, and CUT & TAG (Cleavage under targets and tagmentation) sequencing are used to profile histone modification and TF enrichment. DNase-seq (Deoxyribonuclease I hypersensitive sites sequencing), MNase-seq (Micrococcal nuclease digestion with sequencing), FAIRE-seq (Formaldehyde-Assisted Isolation of Regulatory Elements sequencing), ATAC-seq (Assay of Transpose Accessible Chromatin sequencing), and NOMe-seq (Nucleosome Occupancy and Methylome sequencing) are used to assess chromatin accessibility and nucleosome positioning. DNA methylation arrays and bisulfite sequencing are used to measure global DNA methylation levels. Chromatin interactions are mapped using 3C (chromatin conformation capture), 4C, 5C, Capture-C, Hi-C, DNase Hi-C, Micro-C, ChIA-PET (chromatin interaction analysis by paired-end tag) and HiChIP (Fig. 1c).

In this paper, we aim to introduce methods that are commonly used to map regulatory elements, chromatin accessibility, and chromatin interactions genome-wide. To facilitate researchers who are new in either molecular or computational biology, we describe wet lab as well as dry lab protocols for each of the methods. In particular, we detail chromatin conformation interaction methods and analysis tools, which are relatively new. We also discuss the advantages and limitations of each method and introduce recently developed single-cell based methods. Furthermore, we list currently available 3D epigenomic data sets that are generated in various human cell types and tissues. Introduction of epigenomic methods and resources described here will assist many researchers in the design and analysis of their research projects.

## Main text

### Methods to map regulatory elements

The advancement of molecular biology techniques and next generation sequencing has led to the development of methods to identify regulatory elements throughout the entire genome by analyzing protein-DNA interaction, histone modification, chromatin accessibility, and DNA methylation. The enrichment of specific DNA-binding protein and histone modification is used to identify regulatory elements. Chromatin accessibility analysis reveals open and closed chromatin regions and nucleosome positioning. DNA methylation studies identify the location of methylated CpG sites, which is used to infer chromatin states of regulatory elements and their influences in gene expression [16]. As different factors (protein-DNA interaction, histone modification, chromatin accessibility, and DNA methylation) are assayed, various size of regulatory elements can be identified depending on the methods [17]. Here we introduce commonly used methods to map regulatory elements.

### Assays for protein-DNA interaction and histone modification

**Fig. 2:**
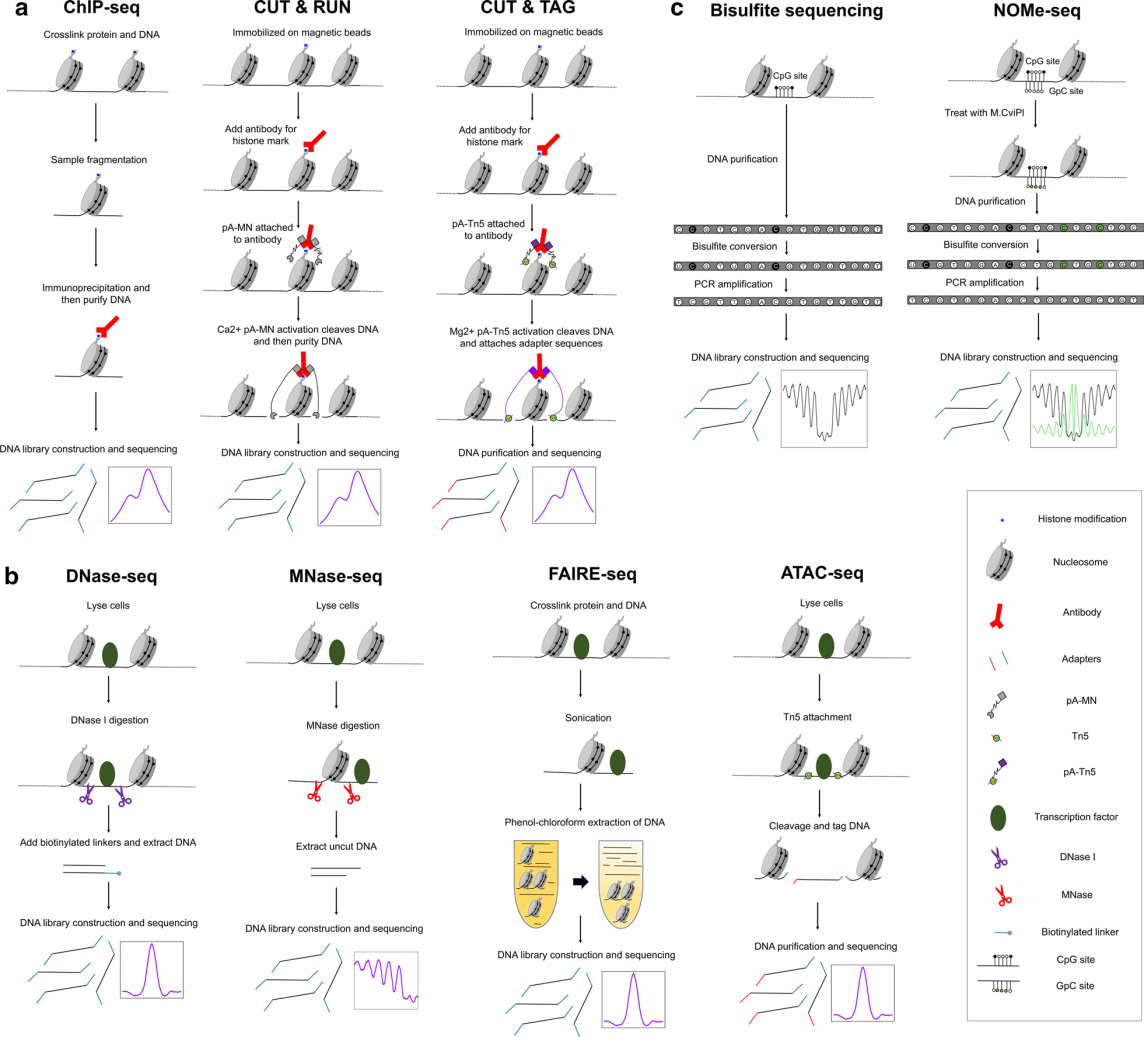
Methods to map regulatory elements. **a** Simplified protocols of methods to identify regulatory elements using histone modifications are shown. ChIP-seq is performed in lysed cells, while CUT & RUN and CUT & TAG are performed in intact nuclei. **b** Simplified protocols of methods to map chromatin accessibility are shown. Cells are lysed, and the DNA is either fragmented with enzymes or through sonication. **c** Simplified protocols of methods to measure global DNA methylation levels are shown. Bisulfite sequencing uses bisulfite conversion followed by sequencing. NOMe-seq simultaneously detects endogenous DNA methylation levels (CpG) and chromatin accessibility (GpC). Bisulfite treatment converts unmethylated C into U, which is converted to T during PCR amplification. pA-MN: Protein A and micrococcal nuclease. pA-Tn5: Protein A and Tn5 transposase, M.CviPl: GpC Methyltransferase

ChIP-seq [18] is one of the popular methods to analyze protein-DNA binding or histone modifications (Fig. 2a). Because regulatory elements are marked by specific proteins that bind to DNA and histone modifications, ChIP-seq has been utilized to profile the activities of regulatory elements [19]. ChIP-seq wet lab protocol includes following steps. First, to identify the regions occupied by TFs or marked by histone modifications, cells can be fixed using crosslinking reagents such as formaldehyde. To localize histone modifications and nucleosome positioning, native ChIP can be done without crosslinking [20]. Second, nuclei are isolated from cells using lysis buffer. Third, the DNA is sonicated or enzymatically fragmented to produce sheared chromatin and quantified for the next step to capture specific regions of interest. Fourth, the sheared chromatin is immunoprecipitated with an antibody specific to the protein or histone modification of interest. Next, the DNA–protein complex is separated by reverse crosslinking as needed. Finally, the pulled down DNA is purified to generate a library by adding adapters for sequencing. The library is sequenced to determine the global genomic regions bound by the protein or marked by the histone modification [21].

ChIP-seq bioinformatic pipeline includes (1) mapping of sequenced reads to the genome, (2) quality check (QC) of sequenced data sets, (3) calling peaks to identify TF binding sites or histone mark enriched regions, and (4) downstream analysis steps to characterize TF binding sites or identify regulatory elements. First, the sequenced reads (e.g., fastq files) are aligned to the reference genome (e.g., human genome assembly 38 (GRCh38) a.k.a. hg38) using mapping software such as BWA [22] or Bowtie2 [23]. Second, the quality of ChIP-seq data sets is checked. To remove poorly sequenced reads, PCR duplicated reads, and unaligned reads, sequenced reads are filtered using programs such as FastQC [24], Picard [25], bedtools [26], or Samtools [27]. The quality of ChIP-seq data sets is further checked by calculating quality metrics such as PCR Bottleneck Coefficient (PBC), Non-Redundant Fraction (NRF), Normalized Strand Cross-correlation coefficient (NSC), and Relative Strand Cross-correlation coefficient (RSC) [28]. These quality control and filtering processes are necessary to determine whether the ChIP-seq data sets are of high quality with library complexity (high fraction of DNA fragments that are non-redundant and mapped to genome) and highly enriched signals [29]. Third, using aligned reads from the ChIP sample and input sample, which indicates background signals, significantly enriched genomic regions are called with peak calling software programs [30–32] such as SPP [33] and MACS2 [34]. To reduce technical variation and identify reproducible peaks, it is recommended to perform ChIP using at least two biological replicates. To measure consistency between replicates, metrics such as Irreproducible Discovery Rate (IDR), which identifies reproducible peaks by generating pseudo replicates from true replicates to call and compare peaks, can be also calculated [35]. Finally, for downstream analysis, genomic distributions of called peaks are analyzed to characterize TF binding sites or identify regulatory elements using programs such as HOMER [36] and ChIPseeker [37]. Differential enrichment of ChIP-seq signals between conditions can be evaluated using programs [38, 39] such as DiffBind [40] and MAnorm [41]. Furthermore, enriched TF motifs at identified peaks and regulatory elements can be determined using motif-search programs such as MEME Suite [42], Transfac [43], Jaspar [44], and HOMER [36]. Identified TF motifs by TF ChIP-seq data are archived in databases such as Factorbook [45].

As the traditional ChIP-seq protocol uses sonication to fragment DNA, the resolution of data is not high. Therefore, ChIP-exo, which is a modified ChIP-seq method that uses exonuclease digestion after ChIP, has been developed [46]. ChIP-exo can identify binding locations at single nucleotide resolution with less background signal [46]. To analyze ChIP-exo data sets, the ChIP-seq bioinformatic pipeline can be used. Specialized bioinformatic tools such as MACE and ChExMix have been developed to analyze ChIP-exo data sets [47, 48]. ChIP-seq requires a relatively large number of cells and has a high background noise. Therefore, methods like CUT & RUN sequencing [49] and CUT & TAG sequencing [50] have been developed to compensate for such limitations. Unlike traditional ChIP that uses fixed cells, CUT & RUN and CUT & TAG methods use unfixed permeabilized cells to facilitate the entry of an antibody into the nuclei, where it binds to TF or histone modification. Unlike ChIP that shears DNA and pulls down enriched regions using an antibody, CUT & RUN uses an antibody and pA-MN (protein A and micrococcal nuclease (MNase) fusion protein) to isolate specific protein-DNA complexes. Calcium ion is added to activate pA-MN, which cleaves the DNA on either side of the binding site of the targeted protein or histone modification. The fragmented DNA that diffuses out of the nuclei is extracted and sequenced after making a DNA library [49]. CUT & TAG is similar to CUT & RUN, except it uses pA-Tn5 transposase instead of pA-MN. pA-Tn5 transposase gets activated by magnesium and ligates an adapter sequence during the cleavage process [50]. Advantages of both CUT & TAG and CUT & RUN are low background noise and lower cell input requirement, since only the DNA that binds to the protein of interest is extracted and sequenced [49, 50]. To analyze CUT & RUN and CUT & TAG sequencing data sets, software programs used for ChIP-seq bioinformatic pipeline can be used. Recently, specialized tools such as SEACR [51], CUT&RUNTools [52], and CUT&TAG pipeline [53] have been developed as well.

### Assays for chromatin accessibility and DNA methylation

Chromatin accessibility can be measured to identify active regulatory elements and nucleosome depleted regions (NDRs), where TFs bind (Fig. 2b). Commonly used methods to measure chromatin accessibility include DNase-seq [54], MNase-seq [55], FAIRE-seq [56], ATAC-seq [57], and NOMe-seq [58]. Unlike histone mark ChIP-seq, CUT & RUN, and CUT & TAG methods that identify regulatory elements which are several kb in size, methods to measure chromatin accessibility can identify smaller-sized NDRs [17]. Moreover, nucleosome and TF footprints can be examined using these methods. These methods do not require an antibody, since they do not target specific proteins or histone marks, so the analysis is not confined to specific TFs or histone modifications [59]. This is advantageous especially when antibodies of the proteins of interest that work for immunoprecipitation and ChIP are not available.

DNase-seq utilizes the Deoxyribonuclease I (DNase I) enzyme that digests accessible DNA regions. Therefore, DNase I hypersensitivity sites (DHS) identified by DNase-seq include open chromatin regulatory regions, where TFs bind [60]. DNase-seq wet lab protocol includes following steps [54]. First, nuclei are isolated from cells using lysis buffer in a similar fashion as ChIP-seq protocol. Second, nuclei are digested using DNase I. DNA fragment sizes are measured to identify optimal digestion using gel electrophoresis. Third, biotinylated linkers are ligated to the ends of digested DNA after polishing to make blunt ends, and the DNA is isolated. Fourth, the DNA with biotinylated linker is digested by restriction endonuclease MmeI and captured by streptavidin-coated Dynabeads to generate short tags to which the second sequencing adaptor can be ligated. Finally, a second linker is ligated and amplified to generate a library for sequencing [54]. Protocols of DNase I digestion and size selection steps may vary by research groups [54, 61, 62]. DNase-seq bioinformatic pipeline is similar to that of ChIP-seq. First, sequenced reads are aligned to reference genome with BWA [22] or Bowtie2 [23] Second, quality of DNase-seq data sets are checked. Poorly sequenced reads, PCR duplicated reads, and unaligned reads are filtered using programs such as FastQC [24], Samtools [27], or Picard [25]. Signal Portion of Tags (SPOT) is used to measure signal-to-noise levels in the genome [63]. Third, the aligned reads are used to call DHS peaks against input sample (background signal) with programs like Hotspot2 [63] or MACS2 [34]. With high-depth sequencing, DNase I cleavage sites can be revealed at base-pair resolution, revealing the presence of TF protected DNA sequences as footprints [64]. CENTIPEDE [65] and DNase2TF [66] are examples of programs that detect these footprints. While DNase-seq shows a greater sensitivity for regulatory sites, especially promoters [67], DNase-seq suffers from sequence specific cutting bias of DNase I that can complicate genomic footprinting [68].

MNase-seq determines chromatin accessibility with micrococcal nuclease (MNase) that preferentially digests nucleosome-free, protein-unbound DNA regions [55, 69]. MNase-seq wet lab protocol includes following steps [70]. First, nuclei are isolated from either native or crosslinked chromatin similar to ChIP-seq protocol. Second, nuclei are digested using MNase with titration. Usually, three to five test digestions with a broad range of total units of MNase is added for a single experiment to help identify the amount of MNase needed for optimal digestion. Third, the uncut DNA is purified and mononucleosome bands are isolated and excised through gel electrophoresis. Finally, the isolated DNA is amplified by adding adapters to generate a library, and sequenced [55]. MNase-seq primarily sequences regions of DNA bound by histones or other proteins [71]. Therefore, it indirectly determines which regions of DNA are accessible by directly determining which regions are bound to nucleosomes or proteins [70]. It is noted that MNase prefers to cut AT-rich sequences in limiting enzyme concentrations [72–74], so careful enzymatic titrations are required for generating accurate and reproducible MNase-seq data sets. While MNase-seq follows most of the software used by DNase-seq for the bioinformatic pipeline (mapping, QC, calling peaks, and downstream analysis), DANPOS2 [75, 76] is reported to be optimized to identify NDRs and dynamic nucleosomes from MNase-seq data sets. Computational analysis with MNase-seq has been also used to predict chromatin interaction and structure [77, 78].

FAIRE-seq is a method, which simply isolates NDRs from chromatin, not using an antibody to target histone mark or TF [56]. FAIRE-seq wet lab protocol includes following steps [79]. First, cells are fixed using formaldehyde so that TFs and histones are crosslinked to interacting DNA like ChIP-seq protocol. Second, crosslinked chromatin is sheared by sonication that generates protein-free DNA and protein-crosslinked DNA fragments. Third, protein-free DNA is isolated using a phenol–chloroform extraction; DNA crosslinked with protein stays in organic phase, while protein-free DNA stays in aqueous phase. Finally, the purified DNA, which includes NDRs, is amplified using adapters to generate a library and then sequenced [56]. The FAIRE-seq bioinformatic pipeline is similar to the DNase-seq pipeline (mapping, QC, calling peaks, and downstream analysis). FAIRE-seq peaks are often called using software such as F-Seq [80], ChIPOTle [81], Mixer [82], or MACS2 [34]. Because FAIRE-seq does not require single-cell suspension or nuclear isolation, it is more adaptable for tissue samples [56]. FAIRE-seq is relatively free from the sequence-specific cleavage bias that is seen in DNase-seq or MNase-seq [59]. However, FAIRE-seq has a higher background level and a lower signal-to-noise ratio, compared to other chromatin accessibility assays, which can limit identifying all open chromatin regions in a given cell [83, 84]. It is reported that FAIRE-seq has lower resolution in identifying open chromatin regions at promoters but captures more distal regulatory elements, compared to DNase-seq [79, 84, 85].

DNase-seq, MNase-seq, and FAIRE-seq require a relatively large number of cells and have high background noise level. Therefore, ATAC-seq was developed to supplement. ATAC-seq uses hyperactive Tn5 transposase that preferentially cuts accessible chromatin regions and simultaneously inserts adapters to the fragmented region [57]. ATAC-seq wet lab protocol includes following steps [57]. First, nuclei are isolated from cells using lysis buffer. Second, Tn5 transposase is added to nuclei, and often cases, detergents such as digitonin, NP40, and Tween-20 are added together in this step to improve cell permeabilization and remove mitochondria from the transposition reaction [86]. Third, DNA is isolated and purified. Finally, fragmented and tagged DNA by Tn5 transposase is purified and then amplified to generate a library and sequenced for analysis. The first step of bioinformatic pipeline of ATAC-seq is adapter trimming. Because of adapter sequences that are added during Tn5 transposase activity, programs like Cutadapt [87] and Trimmomatic [88] are used to remove adapter sequences before alignment. Second, the sequenced reads are mapped to the genome after trimming, similar to other methods. Third, the quality of the data sets is evaluated like ChIP-seq and DNase-seq data sets (see above). Finally, ATAC-seq peaks are called using MACS2 [34] or HMMRATAC, which is a peak calling program specific to ATAC-seq that uses a Hidden Markov model to learn the chromatin structure and predict accessible regions [89]. As in DNase-seq, high-depth ATAC-seq data can be used for genomic footprinting, using HINT-ATAC [90] or CENTIPEDE [65]. The advantage of ATAC-seq is that it is relatively fast and requires a low amount of sample inputs compared to other assays, while maintaining similar specificity [57]. However, ATAC-seq data may be contaminated with a high percentage of mitochondrial DNA [91], so it may require some extra procedures to reduce mitochondrial DNA contamination [92]. Omni-ATAC is one of methods that improve mitochondrial DNA contamination by pretreating DNA with DNase I to remove free-floating and to digest DNA from dead cells [86]. Omni-ATAC is also reported to work using archival frozen tissue samples and 50-μm sections, generating fewer sequencing reads that map to mitochondrial DNA.

NOMe-seq is a method to identify NDRs with M.CviPI methyltransferase that methylates cytosine in GpC dinucleotides not protected by nucleosomes or other proteins (Fig. 2c) [58]. Unlike C^m^pG, GpC^m^ in the human genome does not occur naturally in most cell types [93–95]. Therefore, GpC^m^ levels at open chromatin regions can be compared to background signals and determine NDRs. NOMe-seq wet lab protocol includes following steps [17]. First, nuclei are isolated from cells using lysis buffer. Second, nuclei are treated with M.CviPI and S-adenosylhomocysteine (SAM) to methylate accessible GpC sites. Third, M.CviPI treated DNA is sheared using a sonicator, so that DNA fragments can be sequenced in the later step. Fourth, the DNA is treated with bisulfite, which converts unmethylated cytosine to uracil using sodium bisulfite, while methylated cytosine is unaffected. Finally, library is generated using adapters and sequenced. Since NOMe-seq uses bisulfite treatment, besides GpC methylation, endogenous CpG methylation is also measured [17]. Open chromatin is expected to have high levels of GpC^m^ but low levels of C^m^pG. Therefore, NOMe-seq identifies NDRs using the two separate methylation analyses that serve as independent (but opposite) measures, providing matched chromatin designations for each regulatory element [17]. Bioinformatic pipeline of NOMe-seq includes following steps. First, the sequenced reads are aligned to a bisulfite-converted genome using mapping programs such as BSMAP [96], BWA-METH [97], Bismark [98], BS-SEEKER [99], or Biscuit [100]. Second, Picard [25], Samtools [27], and BamToElementEnrichment script from ECWorkflows [101] are used for QC and post-alignment processing to identify high quality and mapped reads. Third, the methylation status of CpG sites and GpC sites are identified using Bis-SNP [102] or Biscuit [100] programs. Finally, NDRs from NOMe-seq are identified with aaRon R package [103], and plots are generated using programs such as Bis-tools [104]. Unlike other assays, NOMe-seq can determine NDRs at single molecular resolution, and it has no bias toward open chromatin regions, since there is no sonication or digestion with enzyme in the step that identifies open chromatin regions [17]; sonication is done after identifying open chromatin regions to fragment DNA for sequencing purpose. However, it is noted that sequencing cost of NOMe-seq, which is based on whole genome sequencing, is more expensive than other assays such as ATAC-seq.

Quantification of DNA methylation level in regulatory elements also helps us to understand the activities of regulatory elements (Fig. 2c) [4]. Active regulatory elements have relatively low levels of C^m^pG, because proteins bound at open chromatin regions block the DNA methyltransferase (DNMT) complex, needed to methylate cytosine in the regions [105]. On the other hand, DNA methylation in regulatory elements such as CpG island promoters leads to gene silencing [106]. The most common method to assess DNA methylation level is to use bisulfite treatment. Depending on the coverage of profiling, reduced representation bisulfite sequencing (RRBS) [107], DNA methylation arrays [108], and whole genome bisulfite sequencing (WGBS) [93] are used. RRBS uses restriction enzyme digestion to produce sequence-specific fragmentation, and it is the method of choice to study specific regions of interest [107]. For genome-wide analyses, most commonly used methods are using Illumina DNA methylation arrays that can target 27,000 (Human Methylation (HM) 27 K BeadChIP) [109], 450,000 (HM 450 K BeadChIP) [110], and 850,000 (Epic BeadChIP) [111] methylation sites across the genome. Unlike arrays that are restricted to probes, WGBS can assess the DNA methylation status of the entire genome, because whole genome sequencing is used after bisulfite conversion [93]. Similar to NOMe-seq, RRBS and WGBS sequenced data are analyzed by bisulfite mapping programs such as BWA-METH [97], BSMAP [96], Bismark [98] and BS-SEEKER [99]. Quality of DNA methylation data sets are checked with Picard [25] and Samtools [27], and methylated regions are identified using programs like MOABS [112] and methylKit [113]. Illumina DNA methylation array data can be analyzed using software such as Illumina GenomeStudio Software, minfi [114], sesame [115], and DMRCate [116].

The processed sequencing data can be visualized in genome browsers like UCSC Genome Browser [117], Integrative Genomics Viewer (IGV) [118], Integrated Genome Browser (IGB) [119], Ensembl Genome Browser [120], or WashU Epigenome Browser [121]. Commonly used file formats for these genome browsers are bam, bigwig, and bedgraph, which show aligned reads and signal intensity of data sets. Files with bed extensions can be also loaded to the genome browsers to visualize peaks. Some genome browsers like UCSC Genome Browser and Ensembl Genome Browser can only be used as the web-based applications, while IGV and IGB can be used from the local desktop. IGV is also now available as web-based application as well. The web-based genome browsers are generally better at importing and exporting sessions, as data sets can be visualized without downloading data to the local desktop and shared between users.

Recently, advanced techniques using single cell sequencing have been developed to better understand heterogeneity of individual cells. For example, single-cell ATAC-seq, which improves the low input requirement of ATAC-seq further by capturing and assaying cells using a programmable microfluidics platform, has been developed [122]. The specificity of single-cell ATAC-seq identifies chromatin accessibility variance among cell populations, and it is useful to identify sets of TFs associated with specific subgroups [123]. Single-cell NOMe-seq has also been developed using fluorescence-activated cell sorting, and it is reported that it can directly estimate the fraction of accessible regions of individual cells [124]. Single-cell WGBS is also performed. For example, single-cell WGBS on human oocytes revealed distinct DNA methylation patterns in three oocyte maturation stages [125]. Currently, a small number of single-cell ATAC-seq, NOMe-seq, and WGBS data sets have been generated, while thousands of data sets have been generated using a population of cells.

### Data sets that mapped regulatory elements

Large consortia such as ENCODE (Encyclopedia of DNA Elements) [126] and REMC (Roadmap Epigenomics Mapping Consortium) [127] profiled global regulatory elements using over one hundred different cell types. The ENCODE consortium is a project that aims to assemble comprehensive lists of functional elements in the human and mouse genome (https://www.encodeproject.org/). From the phase III of the ENCODE project, a registry of 926,535 human and 339,815 mouse candidate regulatory elements is developed [126]. Data sets generated by the ENCODE project include, but are not limited to, histone mark and TF ChIP-seq, ATAC-seq, DNase-seq, FAIRE-seq, eCLIP-seq, RRBS, DNA methylation array, and WGBS. For example, 2039 ChIP-seq data sets that annotate regulatory elements (promoters, enhancers, and insulators), and 2066 open chromatin and DNA methylation data sets from various cell and tissue types have been generated as of October 2020. As part of the ENCODE project, the functional genomics database that stores thousands of experimental data sets is established. The distinguishing feature of the ENCODE database compared to other databases is its filtering capabilities. Its user-friendly interface allows one to filter experimental data according to assay, target of assay, organism, cell and tissue type, and even developmental stage. Moreover, some data sets can be visualized using its own genome browser and other genome browsers such as UCSC and Ensembl. REMC is a consortium that aims to produce data sets of the human epigenomes that include ChIP-seq of histone modifications, chromatin accessibility, DNA methylation, and gene expression data sets for hundreds of human cell types and tissues (http://www.roadmapepigenomics.org/). Unlike ENCODE, REMC only profiles the human epigenomes, and it does not produce TF ChIP-seq data sets including CTCF ChIP-seq data that mark insulators. The REMC database has searching tools and a matrix, which allows a user to search data sets based on experiment, cell-, and/or tissue-type. Moreover, it has options to visualize data sets in the UCSC genome browser.

There are additional consortia that profile the human epigenomes focusing on specific tissues or diseases. For example, PsychENCODE has profiled the epigenomes of brain cells and tissues obtained from patients who suffer from psychiatric diseases [128]. On the other hand, Blueprint project focuses on hematopoietic epigenomes [129], and The Cancer Genome Atlas (TCGA) specializes in cancer (https://www.cancer.gov/tcga). PsychENCODE has generated ChIP-seq, ATAC-seq, and DNA methylation data sets of more than 750 samples (http://www.psychencode.org/). Blueprint project includes histone modification ChIP-seq, DNase-seq and WGBS data sets (https://www.blueprint-epigenome.eu/), while TCGA mainly contains ATAC-seq and DNA methylation arrays to profile the epigenomes of tumors. Over 400 chromatin accessibility data sets and over 12,300 methylation data sets generated by TCGA are available in GDC data portal (https://portal.gdc.cancer.gov/).

With the increased amount of epigenome data sets generated by researchers, multiple epigenome databases have been developed and maintained. The most commonly used database for regulatory elements data sets is GEO (Gene Expression Omnibus) (https://www.ncbi.nlm.nih.gov/geo/). GEO is a public functional genomics database that archives and freely distributes numerous genomic data sets as part of the National Center for Biotechnology Information (NCBI) [126]. While GEO [130] allows some searching and filtering based on organism and sample type, its query and search mechanism is not as intuitive as that of ENCODE or REMC. However, GEO stores by far the largest amount of epigenome data sets that profile regulatory elements as any researchers can deposit data sets. In addition, European Nucleotide Archive (ENA) [131] led by the European Molecular Biology Laboratory—European Bioinformatics Institute (EMBL-EBI) archives functional genomic data sets resulting from biomedical research projects (https://www.ebi.ac.uk/ena/). Moreover, the International Human Epigenome Consortium (IHEC) coordinates the production of epigenomes from healthy and diseased human cells [132] (http://ihec-epigenomes.org/). Currently, IHEC data portal archives thousands of epigenome data sets generated from ENCODE, REMC, Blueprint, Canadian Epigenetics, Environment and Health Research Consortium (CEEHRC), Japan Agency of Medical Research and Development & Core Research for Evolutional Science and Technology (AMED-CREST), Korean National Institute of Health (KINH), and Deutsches Epigenom Programm (DEEP). Individual research groups also maintain databases by collecting and processing epigenome data sets generated and reported by the research community. For example, Cistrome Data Browser [133] (http://cistrome.org/) encompasses TF, histone ChIP-seq and chromatin accessibility data from GEO, ENCODE, and REMC. ReMap2020 database [134] (http://remap.univ-amu.fr/) collects data sets specialized in transcriptional regulators of DNA-binding experiments in *Homo sapiens* and *Arabidopsis thaliana*.

### Methods to map chromatin interactions

The human genome is tightly packed into the nucleus, because the stretched DNA cannot be contained within the cell size. Increasing evidence suggests that chromatin organization and interaction of regulatory elements influence gene regulation and expression. Local chromatin conformation change can also result in human diseases [13, 14, 135–137]. For example, chromatin conformations affect promoter-enhancer interactions. An enhancer that is located hundreds of kb away from the promoter of a target gene can activate or inactivate the target gene by changing chromatin interaction and organization. Moreover, studies on relationship with polycomb repressive complexes (i.e., PRC1, PRC2) [138–140] and cohesin complex (e.g., CTCF, RAD21) that is enriched at chromatin loop anchors [141, 142] support the importance of chromatin organization in epigenome changes. Here we describe commonly used techniques to profile global chromatin interactions.

**Fig. 3:**
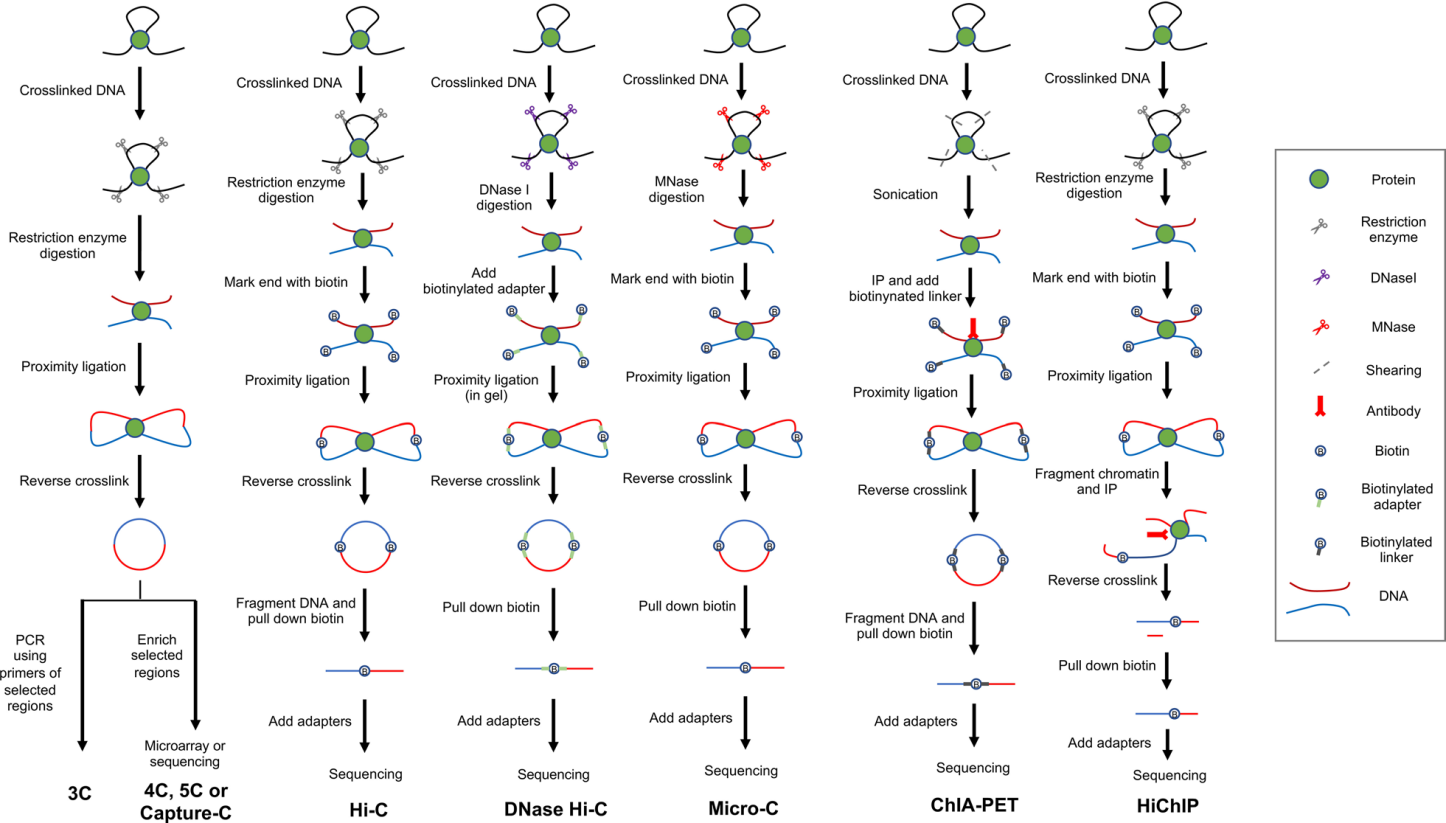
Methods to map chromatin interactions. Simplified protocols of methods to map chromatin interactions are shown. DNA is first crosslinked, and fragmented with restriction enzymes (3C, 4C, 5C, Capture-C, Hi-C), DNaseI (DNase Hi-C), or MNase (Micro-C). After fragmentation, biotin is added for all methods except for 3C, 4C, 5C or Capture-C. The DNA then goes through proximity ligation, and reverse-crosslinked. Purification and amplification steps are followed. ChIA-PET and HiChIP use an antibody specific to TF or histone modification to map chromatin interactions associated with the specific TF or regulatory elements. IP: Immunoprecipitation

Chromatin conformation capture (3C) based techniques are one of useful methods to study the chromatin interactions and the spatial organization of the human genome (Fig. 3). The standard 3C protocol includes following steps. First, cells are crosslinked to fix chromatin segments connected by a protein complex. Second, nuclei are isolated from fixed cells, and then chromatin segments are fragmented using a restriction enzyme. Third, the chromatin fragments, which are in spatially proximity, are ligated together. Next, crosslinking is reversed to isolate ligated DNA. Finally, the purified ligation product (3C template) is quantified with PCR, using primers designed for two chromatin segments looped (one vs one) [143]. 3C is not coupled with next generation sequencing, so 3C cannot assess chromatin interactions genome-wide. Therefore, many derivatives of 3C-based methods (e.g., 4C, 5C, Hi-C) to measure chromatin interactions in many to all loci are developed.

Circular Chromosome Conformation Capture (4C) identifies all possible interactions between a locus of interest with other DNA sequences (one vs all) [144]. 4C wet lab protocol includes additional steps after performing 3C. In 4C, the 3C template is digested again with second restriction enzyme. Then, the product is circularized using ligation. Next, an inverse PCR is performed with primers binding outward on the genomic region of interest to identify and quantify fragments that are ligated to the genomic region of interest. Finally, the amplicons are analyzed using microarray or sequencing to capture all interactions of the genomic region of interest [145]. In 4C-seq (circular chromosome conformation capture, coupled to high throughput sequencing), inverse PCR is performed with a primer that hybridizes to second restriction enzyme fragment and has overhang sequences that corresponds to adapter sequence used in sequencing [145, 146]. 4C-seq bioinformatic pipeline includes following steps. First, the sequenced reads that include the genomic region of interests are kept by demultiplexing and trimmed to extract the sequence including restriction enzyme motifs. Second, data are mapped to reference genome using Bowtie [147] or Novoalign [148]. Third, reads that are mapped to restriction fragment ends with captured regions are quantified using in silico digested reference genome [145]. Finally, read counts are normalized and smoothened, and analyses are performed to identify statistically significant chromatin interactions. Programs like peakC [149], 4C-ker [150], fourSig [151] and FourCSeq [145] are commonly used to identify chromatin interactions from 4C.

Carbon Copy Chromosome Conformation Capture (5C) detects interactions between all restriction fragments within given regions (many vs many) [152]. 5C wet lab protocol includes additional steps after generating the 3C template. To make a 5C library, the 3C template is first converted using multiplex ligation-mediated amplification (LMA), which detects and amplifies specific genomic regions of interest using primer pairs that anneal next to each other on the same DNA strand; In 5C, two sets of primers (5C forward and 5C reverse primers) are annealed to the specific target sequences, and only sequences with both primers attached to the same DNA strand are ligated. The generated 5C library is then followed by microarray or sequencing. For sequencing, universal PCR primers that anneal to tails of 5C primers are used to amplify 5C library for sequencing [152]. The 5C bioinformatic pipeline is similar to 4C. First, paired-end reads are aligned to a pseudo-genome that include all 5C primer sequences using Bowtie [147] or Novoalign [148]. Next, 5C interactions are counted when both paired-end reads are uniquely mapped to the 5C primer pseudo-genome. During this step, invalid interactions that include reads with the same primer or primers of the same type were removed or flagged. Finally, interaction contact matrices are generated using valid interaction counts and normalized for distance and background signals using statistical methods such as quantile normalization [153, 154]. Software such as HiFive [155] and my5C [156] have been developed for 5C data analysis. HiFive is capable of mapping, filtering, normalizing, and visualizing 5C as well as Hi-C data sets, allowing users to analyze the data with a single program [155].

### Assays to map chromatin interactions genome-wide

Unlike 3C, 4C, and 5C, Hi-C can map all possible chromatin interactions across the genome (all vs all) [135–137, 157–159]. Hi-C wet lab protocol includes following steps. First two steps are similar to 3C protocol. First, cells are crosslinked like 3C. Second, nuclei are isolated and then chromatin segments are fragmented by a restriction enzyme. Third, after DNA fragmentation, biotin-labeled nucleotides are added to mark the end. Fourth, segments in proximity are ligated using a DNA ligase. Biotin-label allows enrichment of crosslinked ligation products across the genome. Fifth, the ligated products are reverse-crosslinked. Next, ligation products are fragmented using a sonicator and then pulled down using biotin to generate the biotinylated DNA suitable for sequencing. Finally, by adding adapters needed for sequencing, DNA is amplified and purified. The Hi-C library is then sequenced using paired-end sequencing. By mapping the pair of sequences cut by restriction enzymes and ligated, individually, all possible pairwise interactions between fragments are identified [157].

With the increasing popularity of Hi-C experiments, numerous Hi-C analysis bioinformatic tools have been recently developed. Hi-C bioinformatic pipelines include (1) matrix generation, (2) topologically associating domains (TAD) calling, (3) loop calling, and (4) reproducibility and differential analysis steps (Table 1). Once Hi-C data is generated, the resulting sequencing FASTQ files are first processed to generate a matrix that includes chromatin contact frequencies throughout the entire genome. Examples of matrix generation software include HiC-Pro [160], Juicer [161], Hiclib [162], and Distiller [163] (Table 1). In the first step of matrix generation, read-pairs are aligned to the human genome. During this process, programs account for chimeric reads that span the ligation junction and restriction enzymes that were used. After alignment, the reads are filtered to remove technical artifacts such as PCR duplicates or low-quality alignment reads. Invalid pairs, which are generated due to invalid ligation like dangling end or self-circle circulation, are also filtered. Next, the reads are then mapped through ‘binning’, in which the genome is partitioned into fixed size called ‘bin’, and the number of contacts between bins are assessed and normalized.

**Table 1:** Analysis tools for Hi-C data

Software	Function	How to run	Input	Output
Distiller [163]	Matrix generation	Python package/Linux command line	.fastq	.mcool and .cool
HiCExplorer [214]	Matrix generation	Linux command line	.sam, .bed	.cool or .mcool
Hiclib [162]	Matrix generation	Linux command line	.fastq	.hdf5
HiC-Pro [160]	Matrix generation	Linux command line	.bed	.matrix
HiCUP [215]	Matrix generation	Linux command line	.fastq	.bam
HOMER [36]	Matrix generation	Linux command line/Mac/Windows	.fastq	.txt with matrix information, or .hic
Juicer [161]	Matrix generation	Linux command line	.fastq	.hic
TADbit [216]	Matrix generation	Python package/Linux command line	.fastq, .dsrc	.map
Arrowhead [141]	TAD calling	Linux command line	.hic	.bedpe
CaTCH [176]	TAD calling	R package	.hic	A list of data frame with domain information in R
Domaincaller (Directionaliy Index) [169]	TAD calling	Linux command line	.cool	.bed and .bedgraph
HiCDB [217]	TAD calling	R package/Linux command line with MATLAB	*N* x *N* matrix, or *K* × 3	.txt
HiCseg [175]	TAD calling	R package	Data in r matrix format	List of t_hat, J, and t_est_mat data in R
HOMER [36]	TAD calling	Linux command line/Mac/Windows	HiC tag directory from HOMER	.2D.bed
TADbit [216]	TAD calling	Python package/Linux command line	.bam or .map	List of TADs in Python
TADCompare [218]	TAD calling	R package	N x N matrix, x (N + 3) matrix, or 3-column matrix	Data frame in R
TADPole [219]	TAD calling	R package	Tab-separated matrix file	TADpole object in R
TADtree [220]	TAD calling	Linux command line through Python script	Contact matrix without column or row labels	.txt files with three columns for contact domain information
TopDom [174]	TAD calling	R package	Tab-separated matrix file with bin information	.binsignal and.domain
3DNetMod [221]	TAD calling	Linux command line through Python script	.bed and tab-separated matrix file	Genomic coordinates and statistics output files
FitHiC [179]	Loop calling	R pacakge/Linux command line with Python script	Fragment file and interaction file	.txt
FitHiC2 [180]	Loop calling	Linux command line	Fragment file and matrix file	.txt
GOTHiC [178]	Loop calling	R package	.bam or bowtie2 alignment output	Data frame in R
HiCCUPS [141]	Loop calling	Linux command line	.hic	.bedpe
HiC-DC [222]	Loop calling	Linux command line through R script	File with Hi-C covariates and Hi-C counts	File with data.table and fit information
HiCNormCis (FIREcaller) [223]	Loop calling	R package	N x N matrices file	Object in R
HOMER [36]	Loop calling	Linux command line/Mac/Windows	HiC tag directory from HOMER	.2D.bed
Mustache [182]	Loop calling	Linux command line	.hic, .cool, or raw contact map with normalization vector .txt file and bias .txt file	.tsv file with contact domain information and mustache scale
SIP [181]	Loop calling	Linux command line	.hic, .mcool, or processed file with normalized value	Loop file that can be loaded into Juicebox
StripeCaller [224]	Loop calling	Linux command line	.cool	.bedpe
HiCRep [184]	Reproducibility testing	R package	Squared matrix, 3-column matrix, .hic or .cool	Object in R
HiC-Spector [185]	Reproducibility testing	Linux command line for both Julia and Python implementation	.hic or text delimited matrix file	Reproducibility score
IDR2D [183]	Reproducibility testing	Linux command line	.hic or .matrix and.bed from HiC-Pro	IDR and significance value
FIND [187]	Differential analysis	R package	Three column tab-separated file with matrix and four-column tab-separated file, or .hic	Object in R
HiCCompare [186]	Differential analysis	R package	.hic, .cool, or .matrix, and .bed from HiC-Pro	Object in R
Selfish [188]	Differential analysis	Linux command line	.hic, .cool, .bed, or .matrix	Matrix of q-values in binary numpy file

Hi-C contact matrices often contain systemic biases that can affect the consistency and analysis of the data sets. Therefore, after Hi-C data sets are mapped, the contact matrices are normalized to remove biases such as GC content, mappability, copy number variations, and fragment length (Table 2). The normalization method can be divided into two categories: implicit and explicit. The explicit normalization assumes specific sources of biases and utilizes additional information like fragment length, mappability score, and GC content to correct biases [164]. Examples of software that normalize using the explicit methods include Hicpipe [165] and HiCNorm [164]. On the other hand, the implicit normalization assumes no known source of bias and assumes that all loci have equal representation when there is no bias. Examples of implicit normalization method include ICE (Iterative Correction and Eigenvector Decomposition) [162] and SCN (Sequential Component normalization) [166]. ICE collectively normalizes bias affecting experimental visibility through iterative correction, while SCN normalizes circulation biases. Moreover, there are additional normalization software for other biases such as calCB [167] that normalizes genomic DNA copy number bias in tumor cells and multiHiCcompare that normalizes across multiple data sets [168].

**Table 2:** Normalization tools for Hi-C data

Software	Function	How to run
Binless [225]	Normalize in resolution-agnostic way and adapt to quality and quantity of available data	R package
hicapp/caICB [167]	Normalize genomic DNA copy number bias in tumor cells, as well as fragment length, GC bias, and mappability	Linux command line
HiCorr [226]	Normalize GC bias, mappability, fragment explicitly, and visibility implicitly	Linux command line
HiFive [155]	Normalize through binning, matrix-balancing, and multiplicative-probability model	Linux command line/Python package
HiCNorm [164]	Explicitly normalize fragment length, GC bias, and mappability	Linux command line through R script
Hicpipe [165]	Explicitly normalize fragment length, GC bias, and mappability	Linux command line
multiHiCcompare [168]	Normalize across multiple Hi-C datasets	R package
oneD [227]	Normalize copy number variation bias, especially for biological samples with aberrant karyotype	R package

Mapping genome-wide chromatin interactions by Hi-C and other 3C-derived methods revealed that the human genome consists of compartments and smaller sub-parts. A normalized Hi-C matrix at 1 Mb resolution revealed a plaid pattern, suggesting that chromosome is decomposed into two compartments: compartment A and compartment B [157]. The sequences in compartment A are more closely related with open, accessible, and actively transcribed chromatin, while the sequences in compartment B are more related with closed, inactive chromatin. Compartment A and B partition are cell type-specific, and can be further broken down into sub-compartments, such as A1–A2 and B1–B3 [141]. High-resolution chromatin contact maps revealed highly self-interacting regions that preferentially interact within the domain, and they were referred to as topologically associating domains (TADs) [154, 169, 170]. TADs are suggested to be fundamental components of genome organization as TADs are reported to be conserved across cell types and tissues [171, 172] although recently developed higher resolution of chromatin contact maps revealed that smaller-size TADs (sub-TADs) can vary among cell types [13, 173]. Programs and software such as DI [169], TopDom [174], HiCseg [175], CaTCH [176], and arrowhead [141] have been developed to identify and analyze TADs (Table 1). A previous study has shown that each TAD calling software comes with its own advantages and disadvantages due to their difference in algorithms [177]. Additionally, it is reported that one program can identify TADs that are different in sizes when the bin size of the matrix used to call TADs is changed [177].

Hi-C data sets can be further used to identify chromatin loops [141]. The chromatin loops that have significantly higher contact frequencies, compared with their neighbors were identified as peaks in the Hi-C contact matrix. Examining chromatin loops at higher resolution enables us to study the looping of regulatory elements such as promoter-enhancer loops. Examples of loop calling software include HiCCUPS [141], GOTHiC [178], FitHiC [179], FitHiC2 [180], SIP [181], and Mustache [182] (Table 1). Interaction frequency is compared to the local or global background to determine its significance. Programs like GOTHiC [178], FitHiC [179], and FitHiC2 [180] utilize global background to identify loops, while programs like HiCCUPS [141], SIP [181], and Mustache [182] utilize local background to detect loops. Global background methods can detect interdomain interactions better than local background methods, while the local background methods can detect more significant loops than global background methods [141, 180, 182].

To compare Hi-C data sets, it is crucial to first measure the reproducibility of the generated data sets. However, common statistical methods like Pearson, Spearman or irreproducible discovery rate are not suitable for Hi-C data sets due to their dimensional nature. Therefore, slightly modified methods compatible for Hi-C experiments such as IDR2D [183] and HiCRep [184] are developed (Table 1). IDR2D expands from one-dimensionality of IDR and analyze interactions in two dimensions by a pair of genome coordinates. HiCRep utilizes stratum-adjusted correlation coefficient, a weighted version of Pearson correlation coefficient. Another program called HiC-spector utilizes spectral decomposition to quantify reproducibility of contact maps [185]. After measuring reproducibility of data sets, Hi-C data sets generated in different biological conditions can be further compared to identify regions differentially interacting using programs such as HiCCompare [186], FIND [187], and Selfish [188].

Visualization of Hi-C data sets facilitates data analysis and interpretation. Chromatin contact maps are often represented as a heatmap. In a heatmap, the x-axis and y-axis represent each position along a given chromosome, and each ‘contact’ is represented by a bin, with more frequently interacting contact having stronger color such as dark red, while less frequently interacting contact having weaker color such as white in the white to red color scale. Example software that generate heatmaps include Juicebox [189], HiGlass [190], HiCPlotter [191], HiTC [192], and 3D Genome Browser [193] (Table 3). Heatmaps are sometimes represented as a triangle to facilitate the comparison of Hi-C data sets with other next generation sequencing data sets like ChIP-seq and DNase-seq. Chromatin interactions can be also visualized as loops in genome browsers such as UCSC genome browser [117]

**Table 3:** Visualization tools for Hi-C data

Software	How to run	Input	Interactivity
DNARchitect [228]	Web-based/R package	.bed, .bedpe, .bedgraph, or file with custom headers	Interactive
gcMapExplorer [229]	Linux command line to start GUI	.Gcmap, .ccmap, and .hdf5	Interactive
HiCorr [226]	Linux command line	Anchor-to-anchor looping pairs file from normalization	Non-interactive
HiCPlotter [191]	Python script	.bedgraph, and .matrix from HiC-Pro	Non-interactive
HiGlass [190]	Web-based/Linux command line/Python package	.cool, .mcool, .bigwig, .bedgraphs, and .bed-like	Interactive
HiTC [192]	R package	.matrix from HiC-Pro	Non-interactive
Juicebox [189]	Web-based/local installation on Linux/Windows/Mac	.hic	Interactive
UCSC Genome Browser [117]	Web-based	.hic	Interactive
WashU Epigenome Browser [121]	Web-based	.hic, .cool, .bigInteract, and .longrange	Interactive
3D Genome Browser [193]	Web-based	.butlr	Interactive

Hi-C that uses 6-cutter restriction enzyme fragmentation yields ~ 4 kb fragment size, and even 4-cutter restriction enzyme and multiple restriction enzyme fragmentation results in ~ 1 kb resolution at the best [194]. Therefore, to improve resolution, variations of Hi-C such as DNase Hi-C [195] and Micro-C [194] that use different enzymes to fragment DNA have been recently developed. Unlike Hi-C that uses restriction enzyme to digest crosslinked DNA, DNase Hi-C uses DNase I and Micro-C uses MNase. After digestion, DNase Hi-C includes a step to mark chromatin fragments with biotinylated adapters that contain BamHI restriction enzyme cut sites, instead of simple addition of biotin-marked nucleotides. These sequences are used later to check the DNase Hi-C library size. Unlike Hi-C, where proximity ligation is performed in solution, proximity ligation step for DNase Hi-C is done in gel to reduce random inter-molecular collisions of small-sized DNase-digested fragments [196]. After proximity ligation and reverse-crosslinking steps, DNA can be optionally sonicated for DNase Hi-C if the size of chromatin fragments is too large for sequencing. Micro-C does not require sonication, because MNase already digest DNA to a size less than 500 bp. DNase Hi-C has shown slightly improved resolution over Hi-C, while Micro-C has provided resolution up to ~ 200 bp [194, 195]. While Hi-C bioinformatic pipeline can be used to analyze DNase Hi-C and Micro-C data, difference in fragmentating enzyme needs to be accounted during the steps of mapping and identifying valid pairs to create contact matrices since most Hi-C bioinformatic pipelines utilize restriction enzyme information. To increase the coverage, targeted sequencing methods such as Capture-C [197] and Capture Hi-C [198] that uses oligonucleotide capture technology to enrich the regions of interest are also developed. These targeted sequencing methods can provide enough resolution to identify chromatin contact maps between selected regions of interest while requiring less sequencing depth.

3C methods can be combined with chromatin immunoprecipitation to identify interactions of loci associated with proteins. Chromatin interaction analysis with paired-end tag sequencing (ChIA-PET) [199] combines ChIP and 3C method to detect genome-wide interactions associated with a particular protein. ChIA-PET wet lab protocol includes additional steps after performing ChIP. After performing first four steps of ChIP (crosslinking, nuclei isolation, chromatin fragmentation, immunoprecipitation), biotinylated oligonucleotide half-linkers containing flanking MmeI restriction enzyme sites are added at the ends of DNA. Next, DNA fragments in proximity are ligated like 3C. Furthermore, MmeI restriction enzyme is used to digest to generate a paired end tag (PET) construct, which includes a pair of tags and a linker between the tag pair. Finally, the PET sequences are purified and then PCR amplified for sequencing [199, 200]. MmeI restriction digestion and amplification steps can be alternatively done using Tn5 transposome digestion, in which sequencing adapters are added to the DNA ends simultaneously [201]. ChIA-PET data can be analyzed using bioinformatic tools such as MANGO [202] and ChIA-PET Tool [200] that are specifically designed to process ChIA-PET data sets by filtering linker sequences and mapping to genome to classify PET. Another method called HiChIP [203] is developed to detect interactions associated with proteins of interest. In HiChIP, the restriction enzyme-mediated fragmented DNA goes through in situ proximity ligation like Hi-C, and then immunoprecipitated with a specific antibody of the protein of interest like ChIP. HiChIP is reported to require fewer cells, compared to ChIA-PET [203]. For HiChIP data processing, Hi-C bioinformatic pipeline can be used. HiChIP specialized bioinformatic tools such as hichipper [204] and FitHiChIP [205] can be also used to identify significant chromatin loops.

Recently, single-cell Hi-C is developed to analyze genome organization and variability in individual cells. The first single-cell Hi-C relied on physical separation of cells and resulted in low throughput [206]. However, it is reported that combinatorial cellular indexing to single-cell Hi-C led to significant improvement in genome coverage and throughput [207]. Moreover, SPRITE (Split-pool recognition of interactions by tag extension) method, which measures high-order interactions within an individual nucleus but does not use proximity ligation, is developed to identify chromatin interactions [208]. SPRITE is reported to able to detect interactions that occur at larger spatial distances than the interactions found in Hi-C. Besides these, DNA fluorescence in situ hybridization (DNA FISH) that utilizes imaging method allows for the study of chromosomal organization [209].

### Data sets that mapped chromatin interactions

Because chromatin interaction method is a relatively new technique, there are currently few studies that have generated genome-wide chromatin contact maps in human cells. The 4D Nucleome (4DN) consortium [210] aims to develop experimental and computational approaches to study spatial organization of the genome and its effect on gene regulation and other biological functions (https://www.4dnucleome.org/). Currently, 4DN Data Portal encompasses hundreds of experimental data sets, including Hi-C, Micro-C and DNA FISH data. ENCODE [126] has also generated Hi-C, ChIA-PET, 5C, and SPRITE data, but relatively few data sets compared to other data types. Most of the Hi-C data sets that are currently available are from cell lines and cancer cells. Only few studies have focused on tissues from organs [211, 212], and most of them have a small number of read pairs, which cannot identify all chromatin loops but only identifies large TADs (Table 4). Higher genome coverage is recommended to perform comparison analyses between Hi-C data sets and call chromatin loops for regulatory elements [177, 213]. Therefore, additional higher resolution data sets using Hi-C or 3C-derived methods are greatly needed.

**Table 4:** Hi-C datasets generated in human cells

Name of study	Cell type	Average read coverage (Million read pairs)	Database
Lieberman-Aiden et al*.* [157]	Blood cell lines	30	GEO
Dixon et al*.* [169]	ESCs and lung cell lines	700	GEO
Dixon and Selvaraj et al*.* [230]	Blood cell line	800	GEO
Jin and Li et al*.* [231]	Lung cell line	1500	GEO
Le Dily et al*.* [232]	Breast cell lines	200	GEO
Rao and Huntley et al*.* [141]	Blood cell lines	1700	4DN/GEO
Dixon and Jung et al*.* [233]	ESCs and ESC-derived cells	750	GEO
Grubert and Zaugg et al. [234]	Blood cell line	1400	GEO
Leung and Jung et al. [212]	Thymus, aorta, left ventricle, and liver	300	GEO
Sanborn and Rao et al. [235]	Blood cell lines, lung cell line, and breast cell line	100	4DN
Schmitt and Hu et al. [236]	ESCs, ESC-derived cells, heart, spleen, adrenal gland, brain, lung, pancrea, liver, bladder, ovary, muscle, intestine, and blood and lung cell lines	Cell lines: 800 Tissues: 200	GEO
Taberlay and Achinger-Kawecka et al. [237]	Prostate cell line	500	GEO
Won et al. [238]	Cortical tissues	2000	GEO/dbGaP
Fritz and Ghule et al. [239]	Breast cell lines	400	GEO
Haarhuis and van der Weide et al. [240]	Blood cell lines	150	4DN
Phanstiel and Van Bortle et al. [241]	Blood cell line	5000	Bioproject
Rao et al. [242]	Colon cell lines	1000	4DN
Rubin and Barajas et al. [243]	Keratinocyte and derived cells	400	GEO
Li and He et al. [244]	Blood cell line	400	GEO
Lin et al. [245]	Blood cell lines	750	GEO
Vian and Pękowska et al. [224]	Blood cell line	300	4DN
Abramo et al. [246]	Cervix adenocarcinoma cell lines	300	4DN
Gorkin and Qiu et al. [247]	Blood cell lines	700	GEO/4DN
Greenwald et al. [211]	iPSCs and iPSC-derived cells	300	GEO/dbGAP
Johnston et al. [29]	Brain cell lines	900	GEO
Ray and Munn et al. [248]	Blood cell line	200	4DN
Rhie et al. [13]	Prostate cell lines	1000	GEO
Zhang et al. [249]	ESCs and ESC-derived cells	3000	4DN
Akdemir et al*.* [250]	Blood cell lines, lung cell line, and breast cell line	350	GEO

## Conclusions

There have been striking improvements in both molecular and computational methods to analyze regulatory elements over the last decade. Chromatin immunoprecipitation, chromatin accessibility, and DNA methylation assays have annotated regulatory elements and revealed interactions between TFs and regulatory elements. Recently developed 3C-based methods have shown how these regulatory elements interact with each other genome-wide. Moreover, new methods enable further research of regulatory elements and their interactions in single cell and single molecule resolution. Although thousands of epigenomic data sets have been generated up until now, profiling of regulatory elements and chromatin structures in additional normal and diseased cells is in great demand, because 3D epigenetic signatures are distinct among cell types and cell populations. Further identification and characterization of regulatory elements that control transcription in a cell-type specific manner will enlighten novel molecular mechanisms of gene regulation and diseases.

## Data Availability

Not applicable.

## Abbreviations

3C: Chromatin conformation capture
4C: Circular chromosome conformation capture
5C: Carbon copy chromosome conformation capture
4DN: The 4D nucleome
AMED-CREST: Japan agency of medical research and development & core research for evolutional science and technology
ATAC-seq: Assay of transpose accessible chromatin sequencing
CEEHRC: Canadian epigenetics, environment and health research consortium
ChIA-PET: Chromatin interaction analysis by paired-end tag
ChIP-seq: Chromatin immunoprecipitation sequencing
CTCF: CCCTC-binding factor
CUT & RUN: Cleavage under targets and release using nuclease
CUT & TAG: Cleavage under targets and tagmentation
DEEP: Deutsches epigenom program
DNA FISH: DNA fluorescence in situ hybridization
DNase I: Deoxyribonuclease I
DHS: DNase hypersensitivity sites
DNase-seq: Deoxyribonuclease I hypersensitive sites sequencing
DNMT: DNA methyltransferase
EMBL-EBI: European molecular biology laboratory–European bioinformatics institute
ENA: European nucleotide archive
ENCODE: Encyclopedia of DNA elements
FAIRE-seq: Formaldehyde-assisted isolation of regulatory elements sequencing
GEO: Gene expression omnibus
HM: Human methylation
ICE: Iterative correction and eigenvector decomposition
IDR: Irreproducible discovery rate
IGB: Integrated genome browser
IGV: Integrative genomics viewer
IHEC: The international human epigenome consortium
KINH: Korean national institute of health
MNase: Micrococcal nuclease
MNase-seq: Micrococcal nuclease digestion with sequencing
NCBI: The national center for biotechnology information
NDRs: Nucleosome depleted regions
NOMe-seq: Nucleosome occupancy and methylome sequencing
NRF: Non-redundant fraction
NSC: Normalized strand cross-correlation coefficient
pA-MN: Protein A and micrococcal nuclease
pA-Tn5: Protein A and Tn5 transposase
PBC: PCR bottleneck coefficient
QC: Quality check
REMC: Roadmap epigenomics mapping consortium
RRBS: Reduced representation bisulfite sequencing
SCN: Sequential component normalization
SPRITE: Split-pool recognition of interactions by tag extension
SPOT: Signal portion of tags
TADs: Topologically associating domains
TCGA: The cancer genome atlas
TFs: Transcription factors
TSS: Transcription start sites
WGBS: Whole genome bisulfite sequencing
